# Editorial: Data driven and model based computational futures in cardiovascular practice

**DOI:** 10.3389/fcvm.2023.1336340

**Published:** 2023-11-27

**Authors:** R. Bauer, A. F. G. Cicero, M. Manca

**Affiliations:** ^1^ Nature Inspired Computing and Engineering Research Group, Department of Computer Science, University of Surrey, Guildford, United Kingdom; ^2^ Hypertension and Cardiovascular Risk Research Group, Medical and Surgical Sciences Department, University of Bologna, Bologna, Italy; ^3^ Cardiovascular Medicine Uniti, IRCCS AOU di Bologna, Bologna, Italy; ^4^ SCImPULSE Foundation, Geleen, Netherlands

**Keywords:** computational modeling, computational modeling methods, Cardiovascular disease, Cardiovascular medicine, computational biomedical engineering, computational biomedical research

**Editorial on the Research Topic** Data driven and model based computational futures in cardiovascular practice

With the turn of the millenium medicine has been witness to a tidal change in practices around real world evidence, and research, whose opportunity arose from the growing availability of computing resources, and the associated ever increasing ability to collect and store data. The value and perils of this new normal have become evident during the COVID-19 (1).

In this special theme for Frontiers in Cardiovascular Medicine, we set out to collect contributions from the biomedical research concerned with pushing the boundaries of our understanding and ability to operate in digital (cardiovascular) medicine, with a special focus on two apparently alternative approaches: machine learning, and physiological models.

Enthusiasm about the promises of machine learning applications in the biomedical field is evergrowing, pushed by the amazing list of state-of-the-art works established by data-driven computational approaches in recent years (2, 3). Nevertheless, real-world applications are lagging behind, more so as we focus our attention closer on the patients and the final decisions to be taken during care, where problems like clinical validation (or lack thereof) (4), regulatory grey areas, and the challenges of bias moderation hit the hardest. Whilst advocates are especially vocal about data availability being the bottleneck for progress in such areas, significantly driving policy making at scale (5), some obstacles (e.g., challenges on the path to inferring causality from data, the inevitability of the crystallisation of prior biases from data into the learnt models (6), lacklustre design research in communicating uncertainty and supporting evidence/ elements to a human user, …) appear to be not as trivial as the community would often frame them to be.

Interest in hypothesis driven computational model approaches has been growing, and in some notable cases (e.g., Virtual Physiological Human (7) has been able to attract meaningful institutional support. The advantage of models is that they make the domain of existence/relevance and their fundamental assumptions explicit, allowing humans at once to learn from their failures and refinement and to confidently decide when/how to rely on them. Causality inference, and biases, are thus amenable to explicit conversations, and the approach has a more prominent place for humans in the loop compared to alternatives. On the downside though, model-based computational approaches require longer to approach new problems and can extend only as far as our knowledge stretches.

We are proud of the excellent quality work we have been able to attract and include. The manuscripts we could map to model based computational approaches have shown a mature field whose solutions are under active development towards augmenting clinical practice.

Chaudhuri et al. present a computational model providing predictive haemodynamic parameters of functional graft performance to aid surgeons avoiding grafts configurations with have poor flow and patency, which is later tested in a clinical challenge where 16 cardiac surgeons selected their favourite strategy for 5 patients before and after using the computational model in Chaudhuri et al., demonstrating an improvement of decision making in anaortic operations.

Benemerito et al. present a multiscale computational framework to simulate mechanical thrombectomy procedures to provide quantitative assessment of resulting clinically relevant quantities such as flow in the retrieval path. These simulations can be used to find the optimal procedural parameters that are most likely to result in a favorable clinical outcome with the potential to augment clinical evidences in reaching a consensus on procedural parameters such as the use of balloon guide catheters (BGC) to provide proximal flow control.

Reid et al. present the application of virtual imaging technology for diagnosis and planning of the aortic valve sparing procedure demonstrating that obtained information could be successfully applied to pre-operative surgical planning.

The manuscripts we could map to machine learning applications to cardiovascular medicine appeared comparatively competitive on the technological solutions but somewhat less mature in terms of governance and technological solutions available to access the clinical settings.

Cheng et al. present work to develop a reliable prediction tool of composite outcomes of postoperative complications using a minimum of easily accessible clinical parameters.

Jiang et al. present the use of machine learning techniques to identify atrial fibrillation recurrence following Cox-Maze IV procedures.

Baskaran et al. present an exploration of the generalisability of currently available predictors of major adverse cardiovascular events to under-represented populations.

Very interestingly with regards to making machine learning real-world ready, lowering barriers to data access, Cabrero-Holgueras and Pastrana present work to adapt the linear algebra operations of deep learning layers to packed homomorphic encryption demonstrating a speed-up in processing of convolutional layers compared to the existing proposals of privacy-preserving deep learning over encrypted medical data.

We believe this special theme will offer the readers a timely and strategic overview of the state-of-the-art in computational methods applied to cardiovascular medicine, offering a realistic and grounded insight into adjacent possibles they will soon be faced with in research and clinical practice.
